# Phylogenomic Analysis of Velvet Worms (Onychophora) Uncovers an Evolutionary Radiation in the Neotropics

**DOI:** 10.1093/molbev/msab251

**Published:** 2021-08-24

**Authors:** Caitlin M Baker, Rebecca S Buckman-Young, Cristiano S Costa, Gonzalo Giribet

**Affiliations:** 1 Museum of Comparative Zoology, Department of Organismic and Evolutionary Biology, Harvard University, Cambridge, MA, USA; 2 Laboratório de Sistemática e Taxonomia de Artrópodes Terrestres, Departamento de Biologia e Zoologia, Instituto de Biociências, Universidade Federal de Mato Grosso, Cuiabá, Brazil

**Keywords:** biogeography, dispersal, Gondwana, Onychophora, systematics, vicariance

## Abstract

Onychophora (“velvet worms”) are charismatic soil invertebrates known for their status as a “living fossil,” their phylogenetic affiliation to arthropods, and their distinctive biogeographic patterns. However, several aspects of their internal phylogenetic relationships remain unresolved, limiting our understanding of the group’s evolutionary history, particularly with regard to changes in reproductive mode and dispersal ability. To address these gaps, we used RNA sequencing and phylogenomic analysis of transcriptomes to reconstruct the evolutionary relationships and infer divergence times within the phylum. We recovered a fully resolved and well-supported phylogeny for the circum-Antarctic family Peripatopsidae, which retains signals of Gondwanan vicariance and showcases the evolutionary lability of reproductive mode in the family. Within the Neotropical clade of Peripatidae, though, we found that amino acid-translated sequence data masked nearly all phylogenetic signal, resulting in highly unstable and poorly supported relationships. Analyses using nucleotide sequence data were able to resolve many more relationships, though we still saw discordant phylogenetic signal between genes, probably indicative of a rapid, mid-Cretaceous radiation in the group. Finally, we hypothesize that the unique reproductive mode of placentotrophic viviparity found in all Neotropical peripatids may have facilitated the multiple inferred instances of over-water dispersal and establishment on oceanic islands.

## Introduction

Onychophora, commonly referred to as “velvet worms,” is an exclusively terrestrial phylum of invertebrates that lives in humid, dark microhabitats such as rotting logs, leaf litter, and caves. These charismatic animals have attracted the attention of biologists for a multitude of reasons, such as their status as a “living fossil” (Ghiselin 1984; Werth and Shear 2014) and their unique prey-capture and defense mechanism of ejecting glue from oral slime papillae (Benkendorff et al. 1999; Concha et al. 2015). They also have a striking geographic distribution (Monge-Nájera 1995), which recent work has demonstrated is largely the result of ancient Pangaean regionalization and Gondwanan vicariance (Murienne et al. 2014; Giribet et al. 2018).

Onychophora are traditionally united with Arthropoda and Tardigrada in the clade Panarthropoda, one of the major lineages of Ecdysozoa (Aguinaldo et al. 1997; Giribet and Edgecombe 2017). While the internal relationships of Panarthropoda (and even its validity) are still actively debated (Giribet and Edgecombe 2020), many studies have recovered Onychophora as the sister group of arthropods (Hejnol et al. 2009; Rota-Stabelli et al. 2013; Borner et al. 2014; Laumer et al. 2019). As the putative sister group to the most diverse animal phylum, their modest sum of ∼200 described species (Oliveira, Read, et al. 2012) raises macroevolutionary questions about the nature of morphological and genetic radiations and stasis (e.g., Lee et al. 2013). This phylogenetic position also makes them a key lineage for understanding the evolutionary development of arthropods, particularly the so-called “arthropod head problem” (Mayer et al. 2010). But despite the widespread interest in onychophorans across many fields of biology, much of their basic natural history, as well as several aspects of the internal phylogeny, remain poorly understood (Mayer et al. 2015).

**Fig. 1.**
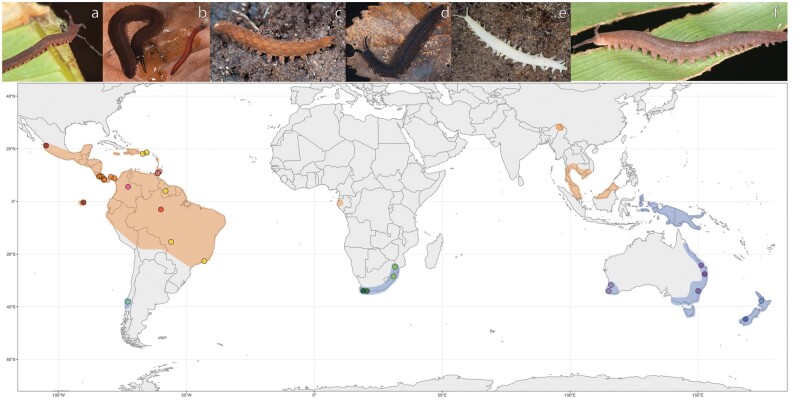
Distribution of samples used in this study, colored by geography and clade identity (see figs 2 and 3). Peripatidae is shown in warm tones and Peripatopsidae in cool tones. General distributions of Peripatidae and Peripatopsidae are indicated by orange and blue polygons, respectively. (*a*–*f*) Live habitus of Onychophora. (*a*) *Epiperipatus* sp. MCZ-131442 displaying the unique prey-capture method of shooting glue out of slime glands. (*b*) *Macroperipatus torquatus* MCZ-143928, a placentotrophic viviparous adult female with a recently birthed juvenile. (*c*) *Ooperipatellus* sp. MCZ-152165. (*d*) *Peripatoides novaezealandiae* MCZ-152262. (*e*) *Peripatopsis alba* (not collected), a troglobitic species. (*f*) *Epiperipatus* sp. MCZ-136557, a rogue taxon in our analyses. Photographs (*a*), (*b*), (*e*), and (*f*) by G.G.; (*c*) and (*d*) by C.M.B.

There are two extant families within Onychophora. The family Peripatidae has a circum-tropical distribution, its 76 valid representatives distributed in Southeast Asia, West Africa, and the Neotropics, the last of which holds the vast majority of the family’s diversity (Barquero González et al. 2020; Giribet and Edgecombe 2020; Costa and Giribet 2021). This family also contains the only fossils that are identifiably members of crown-group Onychophora, from Late Cretaceous Burmese amber (Grimaldi et al. 2002; Oliveira et al. 2016). The other family, Peripatopsidae, has a circum-Antarctic distribution with 111 representatives in Chile, South Africa, and Australasia (fig. 1) (Barnes et al. 2020; Giribet and Edgecombe 2020). The families are supported by a suite of morphological characters (Reid 1996), their validity having been previously confirmed using Sanger DNA sequencing (Murienne et al. 2014; Giribet et al. 2018).

Phylogenetic relationships within these families, however, are in many cases poorly supported or unresolved, particularly within the Neotropical lineage of Peripatidae (Giribet et al. 2018; Costa et al. 2021). Systematic and taxonomic work in the group is stymied by several factors. For one, despite the morphological diversity seen in Cambrian fossils probably belonging to stem-group onychophorans (Yang et al. 2015), extant velvet worms are highly morphologically conserved (Mayer et al. 2015) and have been so since the Carboniferous, where the fossil *Antennipatus montceauensis* (unassignable to the stem- versus crown-group) from France bears multiple onychophoran-specific features (e.g., annulated antennae, slime glands) (Garwood et al. 2016). Because of this limited morphological disparity, taxonomy requires careful examination of characters to differentiate species, often using scanning electron microscopy (SEM) (Ruhberg 1985; Reid 1996; Oliveira, Franke, et al. 2012; Daniels et al. 2016); even so, consistent morphological differences may not be detected (e.g., Sato et al. 2018; Costa et al. 2021). Additionally, a number of monotypic genera have been erected on the basis of autapomorphic characters, but these species have subsequently been found to nest within other genera (Giribet et al. 2018; Costa et al. 2021). In the case of one genus (*Macroperipatus*), its diagnostic trait is even suspected to be an artifact of fixation in ethanol for at least some of its constituent species (Oliveira et al. 2010).

The application of molecular data to onychophoran systematics has been revolutionary, providing increased phylogenetic resolution, particularly within Peripatopsidae, and elucidating a large number of cryptic species (Briscoe and Tait 1995; Tait and Briscoe 1995; Gleeson et al. 1998; Trewick 1998, 2000; Daniels et al. 2009; Allwood et al. 2010; Bull et al. 2013; Oliveira et al. 2013; Murienne et al. 2014; Daniels et al. 2016; Oliveira and Mayer 2017; Giribet et al. 2018; Oliveira et al. 2018; Sato et al. 2018). However, onychophorans have notoriously challenging molecular characteristics, with extremely large nuclear genomes that are suspected to contain many repetitive elements (Jeffery et al. 2012), low GC content (Mora et al. 1994), highly variable mitochondrial genomes showing gene rearrangements and pseudogenes (Podsiadlowski et al. 2008; Braband, Cameron, et al. 2010; Braband, Podsiadlowski, et al. 2010), and novel insertions in variable regions of 18S rRNA (Giribet and Wheeler 2001). Together, these factors make the generation of large, multilocus PCR-based Sanger sequencing data sets onerous as many universal primers fail to amplify DNA and many data sets have considerable amounts of missing data, which contributes to the limited resolution in the group.

To add to these challenges, the animals themselves, with a few notable exceptions, are hard to find, as they live in cryptic environments and in most places exhibit low population densities (Daniels et al. 2016). As such, gathering enough specimens to diagnose inter- or intra-specific differences, or to comprehensively sample across their known geographic and taxonomic spectra, requires extensive collecting efforts.

Over the past decade, we have been fortunate enough to collect and receive donations of onychophoran tissues suitable for RNA sequencing from nearly all the major landmasses from which they are known (fig. 1). While transcriptome sequencing has the drawback of requiring fresh or specially preserved tissue, excluding the utilization of most museum specimens, it is promising for the study of onychophoran systematics because it circumvents the limitations of PCR amplification to generate large amounts of sequence data for each individual. Furthermore, as a reduced-representation genomic sequencing method, it bypasses the need to sequence the extremely large and presumably repetitive nuclear genomes of onychophorans, and its implementation requires no prior genomic knowledge (as is the case with target enrichment methods). Given that transcriptome sequencing has successfully resolved evolutionary relationships across the animal tree of life (e.g., Dunn et al. 2008; Rota-Stabelli et al. 2013; Kocot et al. 2017; Laumer et al. 2019), and within Arthropoda in particular (Misof et al. 2014; Schwentner et al. 2017; Fernández et al. 2018; Sharma et al. 2018; Lozano-Fernandez et al. 2019), we, therefore, sequenced transcriptomes of species from both families of Onychophora in an attempt to clarify evolutionary relationships and biogeographic patterns in the phylum, particularly within the Neotropical clade of Peripatidae—a clade known as Neopatida. As such, this study reflects the best sampling of onychophorans that is currently available for a genomic scale data set.

## Results and Discussion

### Phylogenetic Relationships of Peripatopsidae

**Fig. 2.**
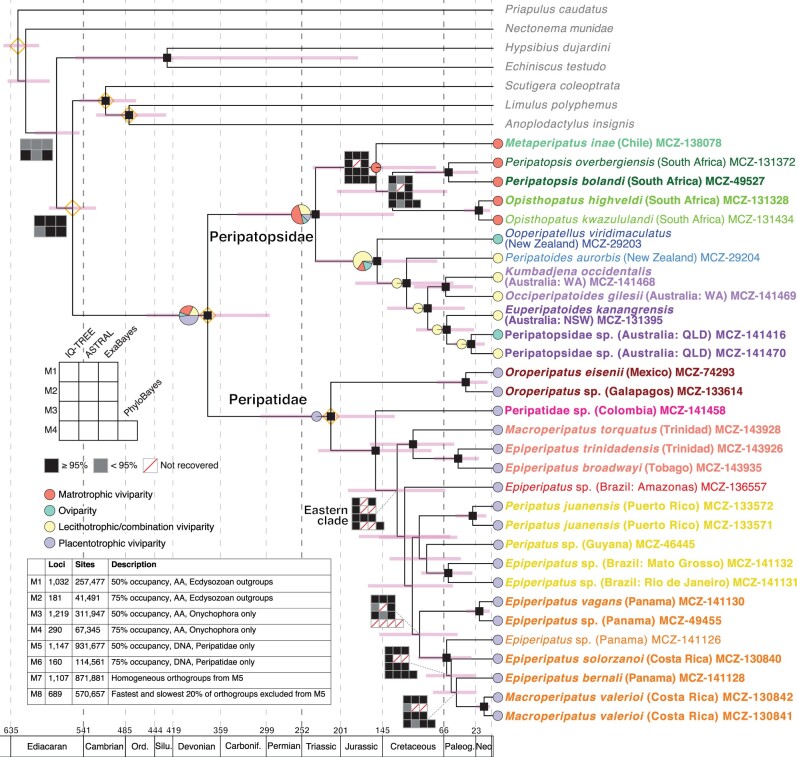
Chronogram of Onychophora (minimum age constrained to 300 Ma) with a summary of phylogenetic relationships inferred from AA data (M1–4). Terminals colored as in figure 1. Small black squares indicate nodes recovered in all analyses with strong support (IQ-TREE ultrafast bootstrap, ASTRAL local posterior probability [PP], ExaBayes PP, PhyloBayes PP ≥ 95%). Support plots were drawn at nodes recovered in >50% of analyses but which did not receive high support across all analyses (outgroup nodes with M1 and M2 results only). Nodes recovered in fewer than 50% of analyses are unlabelled. 95% highest probability density (HPD) intervals for divergence times drawn at nodes in light pink. Orange diamonds denote nodes constrained in divergence time estimation. Reproductive mode indicated by colored circles at terminals, with ancestral state reconstructions (ER model) shown at nodes with pie charts. The inset table gives descriptions of matrices used in phylogenetic analyses.

We recovered the monophyly of Onychophora, as well as the reciprocal monophyly of Peripatopsidae and Peripatidae, with full support across all analyses (fig. 2). This result is consistent with previous analyses that included non-onychophoran outgroups (Murienne et al. 2014; Giribet et al. 2018). We also recovered Arthropoda as the sister group to Onychophora with high support in all analyses (fig. 2; supplementary figs. S1–S6, Supplementary Material online).

Within Peripatopsidae, we recovered a well-supported and stable topology across our treatments. The family was composed of two main clades, one of which contained taxa from South Africa and Chile (landmasses comprising the former West Gondwana) and the other from Australia and New Zealand (East Gondwana), again in line with previous studies (Murienne et al. 2014; Giribet et al. 2018; Oliveira et al. 2018).

The clade corresponding to West Gondwana found a monophyletic South Africa (*Opisthopatus* and* Peripatopsis*) to the exclusion of *Metaperipatus* from Chile in all but one analysis. The deviant result (ASTRAL analysis of M2) instead recovered a sister group relationship between *Metaperipatus* and* Peripatopsis* with marginal support (0.41 local posterior probability [PP], supplementary fig. S5, Supplementary Material online). Our main tree topology contradicts the results of Sanger-based studies that have included these three genera, all of which recovered *Peripatopsis* + *Metaperipatus*, though with variable support (Allwood et al. 2010; Murienne et al. 2014; Giribet et al. 2018). An approximately unbiased (AU) test on M2 comparing the ML tree to the deviant topology (*Metaperipatus* + *Peripatopsis*) also found South African monophyly to be significantly better than the alternative (delta*L* = 88.435, *p*-AU = 4.99e−08). However, we were unable to include representatives of the monotypic Chilean genus *Paropisthopatus*, which has been hypothesized to be closely related to *Opisthopatus* (Daniels et al. 2016), and note that these results could change with its inclusion. Notably, all members of this clade utilize the reproductive mode of matrotrophic viviparity (eggs have little or no yolk and the mother prodives nutrients to embryos, though not via a placenta), a potential synapomorphy for the group (though this is also found in *Paraperipatus*, a genus from New Guinea and Indonesia) (Mayer et al. 2015).

Within the Australian and New Zealand clade, the first lineage to diverge was *Ooperipatellus*, an oviparous genus found in both New Zealand and Tasmania. Previous studies have demonstrated that within this genus, the taxa from those landmasses are reciprocally monophyletic with strong support (Murienne et al. 2014; Oliveira and Mayer 2017; Giribet et al. 2018). The viviparous New Zealand-endemic genus *Peripatoides* was recovered as the next lineage to diverge (we use the general term “viviparous” here when detailed studies have not been conducted to assess how much yolk eggs contain or whether mothers provide nutrients to embryos). Similar to *Ooperipatellus*, previous phylogenetic analyses found that *Peripatoides* is closely related to a suite of viviparous taxa from Tasmania (*Diemenipatus*, *Leucopatus*, *Tasmanipatus*) with strong support (Murienne et al. 2014; Giribet et al. 2018; Oliveira et al. 2018). Interestingly, Murienne et al. (2014) and Giribet et al. (2018) recovered these two *trans*-Tasman Sea clades as each other’s sister group; however, Oliveira and Mayer (2017) and Oliveira et al. (2018) recovered *Ooperipatellus* as the sister group to a clade of mainland Australian taxa, albeit with low support. Our results contradict all of these studies, placing *Peripatoides* (and presumably the viviparous genera from Tasmania) as the sister group to a clade of taxa from mainland Australia.

In the clade from mainland Australia, we found a division between taxa from Western and eastern Australia. The two genera from Western Australia (*Kumbadjena* and* Occiperipatoides*), both of which are viviparous, formed a clade, a result found in many previous analyses based on molecular and morphological data (Reid 1996; Murienne et al. 2014; Oliveira and Mayer 2017; Giribet et al. 2018; Oliveira et al. 2018). We also recovered a clade from eastern Australia, which was represented in our phylogeny by three specimens. Of these, two (*Euperipatoides kanangrensis* and Peripatopsidae sp. MCZ-141470) were viviparous, and the other (Peripatopsidae sp. MCZ-141416) was oviparous. Eastern Australia is home to a great diversity of velvet worms, currently organized into 36 genera with 41 species total (Reid 1996), and as such our sampling is far from comprehensive. We were unable to assign the two specimens from Queensland to genus as taxonomy of the eastern Australian velvet worms is largely based on internal characters or features of the integument, both of which get distorted or destroyed by preservation in RNA*later*. Despite those limitations, our results underscore the evolutionary lability of reproductive mode in Peripatopsidae, finding at least three mode changes within the family (fig. 2). They also highlight the promise of using transcriptomics to sort out the taxonomy in this group so as to better characterize the full extent of Australian peripatopsid diversity. Finally, while we lack representatives of the genus *Paraperipatus* from New Guinea and Indonesia, this genus was found to be the sister group to all other East Gondwanan peripatopsids in Giribet et al. (2018) with full support. That genus may therefore constitute the first diverging lineage in the Australia–New Zealand clade, before *Ooperipatellus*, and future work should test this hypothesis if fresh tissue becomes available. If the position of *Paraperipatus* is confirmed, then matrotrophic viviparity would likely optimize as the ancestral reproductive mode of the Australasian clade.

### Phylogenetic Relationships in Peripatidae

Our sampling within Peripatidae was restricted to the Neotropical clade (Neopatida). However, previous phylogenetic studies have demonstrated that the first two divergences within the family correspond to *Eoperipatus* (from Southeast Asia), followed by the monotypic *Mesoperipatus* (from West Africa), which is then sister group to the diverse clade of Neopatida (Murienne et al. 2014; Giribet et al. 2018). No phylogenetic analysis has yet included the Indian genus *Typhloperipatus*, but it has been proposed to be related to the other Southeast Asian genus (Oliveira et al. 2016). Despite sequencing over 80 Neopatida specimens from across an extensive geographic range, Giribet et al. (2018) found the evolutionary relationships within this clade to be extremely poorly supported, with a few exceptions. They were able to recover the monophyly of taxa from specific islands or smaller continental areas (e.g., Jamaica, Trinidad and Tobago, Puerto Rico, certain species from Costa Rica and Panama), but relationships between those areas were poorly supported. Additionally, they found strong support for an initial divergence in Neopatida between *Oroperipatus* (from west of the Andes, including the Galapagos, and into Mexico) and the rest of the group, followed by a split between a clade from Colombia and Ecuador and the remaining taxa (from Central America, the Caribbean, and South America east of the Andes, hereafter the “Eastern clade”). The recalcitrance of this Eastern clade has been noted by many previous authors as well (Oliveira et al. 2011; Murienne et al. 2014; Oliveira et al. 2015; Cunha et al. 2017; Costa et al. 2021).

Unfortunately, our results only moderately improved resolution in this group. Analyses of amino acid data (M1–M4) were stunningly inconsistent, recovering ten distinct topologies across 13 treatments, usually with low support (fig. 2; supplementary figs. S1–S13, Supplementary Material online). There were some consistently well-supported relationships: we found the same three clades of Neopatida as in Giribet et al. (2018), corresponding to 1) *Oroperipatus*, 2) a lineage from the Colombian Andes (here represented by a single species), and 3) the Eastern clade (from Central America, the Caribbean, and the rest of tropical South America). Within the Eastern clade, we also recovered the monophyly of taxa from specific geographic areas, such as Trinidad and Tobago, Puerto Rico, and (in eight of 13 treatments) Central America (Costa Rica and Panama). Additionally, eight analyses recovered the taxa from Trinidad and Tobago as the sister group to all other Eastern clade members. But beyond that, very few relationships could be discerned with any confidence.

**Fig. 3.**
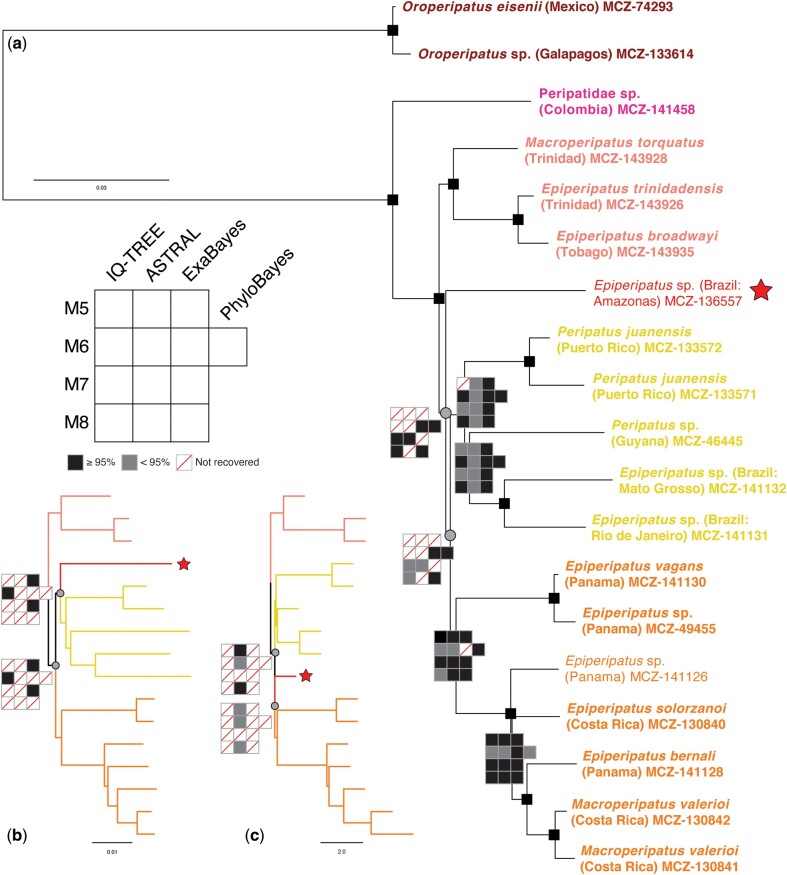
Phylogeny of Neopatida inferred from nucleotide data. (*a*) Summary of relationships mapped onto the topology of PhyloBayes analysis of M6. Small black squares, support plots, and terminal colors as in figures 1 and 2. Star denotes the rogue taxon *Epiperipatus* sp. (MCZ-136557) from Amazonas. Grey circles are drawn at the node leading to the specimen from Amazonas and the clade of composed of (Amazonas + Central America + Puerto Rico–Guyana–Brazil). (*b, c*) Alternative topologies of Neopatida showing possible placements of the sample from Amazonas.

We then generated a third set of matrices that contained only the peripatid taxa. The average pairwise identity of the recovered amino acid orthogroups was very high (94.5%), suggesting that the observed incongruence in this clade could be due to limited phylogenetic signal. We therefore went back to the untranslated nucleotide sequences to perform additional analyses, hoping they would harbor increased signal (fig. 3; supplementary figs. S14–S30, Supplementary Material online). This approach yielded better resolution, universally recovering the clade from Trinidad and Tobago as the sister group to the remaining members of the Eastern clade with full support. We also found support for a clade of taxa from the Brazilian Savanna (Cerrado) (MCZ-14132) and Atlantic Rainforest (MCZ-131131), Guyana (MCZ-46445), and Puerto Rico (MCZ-133572 and MCZ-133571) across all but one treatment (see supplementary fig. S18, Supplementary Material online). Furthermore, the clade from Central America was recovered in 12 of 13 analyses (see supplementary fig. S24, Supplementary Material online, also recovered in eight of the AA analyses). But a specimen of *Epiperipatus* from Amazonas (MCZ-136557, pictured in fig. 1*f*, denoted by a star in fig. 3) was highly unstable, coming out most frequently as the sister group to the Central American and Puerto Rico–Guyana–Brazil clades (fig. 3*a*). However, in three analyses it resolved as the sister group to the Puerto Rico–Guyana–Brazil clade alone (fig. 3*b*), and in three other analyses it was the sister group to the Central American clade alone (fig. 3*c*). Furthermore, when this specimen was excluded from ML analyses, we universally recovered the Puerto Rico–Guyana–Brazil clade with strong support, as well as the Central America clade in all but one analysis (supplementary figs. S17, S22, S26, and S30, Supplementary Material online).

### Interrogating the Position of *Epiperipatus* sp. from Amazonas

Given the instability of *Epiperipatus* sp. from Amazonas, we performed a quartet likelihood mapping analysis using all the orthogroups in M5 to determine whether our data were capable of resolving between these three different phylogenetic hypotheses (supplementary fig. S31, Supplementary Material online). We found that almost all quartets (99%) showed strong resolving power (i.e., fell in one of the corners of the triangle), and a slight majority (50.5%) supported a topology in which the specimen from Amazonas was the sister group to the Central American and Puerto Rico–Guyana–Brazil clades (consistent with the topology of fig. 3*a*). However, one-third of the quartets instead supported the topology of figure 3*b*, in which the Amazonas specimen was the sister group to only the clade from Puerto Rico–Guyana–Brazil, obfuscating any clear placement for this species.

Furthermore, a SuperQ network of M5 gene trees (supplementary fig. S32, Supplementary Material online) showed a predominantly radial pattern, displaying high levels of reticulation between the well-supported clades in our phylogeny and indicating extensive gene tree conflict. In particular, the specimen from Amazonas showed no strong affinity to any of the other taxa, substantiating its rogue behavior across our different phylogenetic treatments. This instability was not attributable to missing data, as it was represented in no fewer than 73% of orthogroups across all matrices (supplementary table S3, Supplementary Material online). Also, while this specimen only had 38.5% of complete BUSCOs (supplementary table S1, Supplementary Material online), BUSCO scores were generally low across Peripatidae (family average = 41.8%), and other taxa with lower BUSCO scores and higher amounts of missing data were reliably placed (e.g., *Oroperipatus* sp. Galapagos, both specimens of *Peripatus juanensis*). This star-like supernetwork may therefore reflect a history in which peripatids rapidly radiated across the Neotropics, a scenario that could explain the genetic (and morphological) similarity of Neopatida (as measured by orthogroup pairwise identity), the extremely short basal internodes in the clade, and our overall difficulty resolving relationships.

### Biogeographic Patterns in Peripatopsidae

Our divergence dating analysis with Onychophora constrained to a minimum age of 300 Ma found crown Onychophora to be 376 Ma (95% highest probability density [HPD] 294–457 Ma) (fig. 2). Divergence times from the analysis with no age constraint on the phylum were very similar across the entire tree (supplementary fig. S33, Supplementary Material online). Peripatopsidae was found to be 234 Ma (95% HPD: 131–337 Ma), corresponding to the split between the South Africa–Chile clade and the Australia–New Zealand clade. This mean age predates the initial breakup of the former supercontinent Gondwana into West and East Gondwana ca. 170 Ma (McLoughlin 2001; Ali and Aitchison 2008), though the 95% HPD does overlap with that event. Additionally, the divergence between *Metaperipatus* from Chile and *Opisthopatus* + *Peripatopsis* from South Africa was estimated at 154 Ma (95% HPD: 75–237 Ma), coinciding with the opening of the southern Atlantic Ocean ca. 140–130 Ma (McLoughlin 2001; Eagles 2007). This parallels Gondwanan vicariant patterns seen in previous onychophoran studies (Murienne et al. 2014; Giribet et al. 2018) as well as in other groups of animals such as harvestmen (Baker et al. 2020; Derkarabetian et al. 2021), sphaerodactyl geckos (Gamble et al. 2008), caecilians (Kamei et al. 2012), and potentially water scavenger beetles (Toussaint et al. 2017).

Because we only had one exemplar from each of the New Zealand genera, and also did not have representatives of their Tasmanian counterparts, our chronogram gave little insight into the biogeography of those taxa. However, the estimated divergence times for both *Ooperipatellus and Peripatoides* from their sister clades predated the separation of New Zealand from Eastern Gondwana ca. 80 Ma, consistent with previous studies (Allwood et al. 2010; Murienne et al. 2014; Giribet et al. 2018). Finally, within the Australian clade, we recovered an estimated divergence time between Western and eastern Australia in the mid-Cretaceous, about 86 Ma (95% HPD: 38–140 Ma). This reflects an ancient separation that may be attributable to the formation of an extensive seaway across central Australia in the Cretaceous (McLoughlin and Kear 2010). Divergences within eastern Australia for the most part predated its Miocene aridification (Martin 2006), a process hypothesized to have driven diversification in other Australian taxa (e.g., Rix and Harvey 2012). However, the 95% HPD for the divergence between the two unidentified specimens of Peripatopsidae from Queensland does fall within the Miocene (mean: 29 Ma, 95% HPD: 11–52 Ma). The inclusion of additional taxonomic and geographic diversity from eastern Australia will therefore be critical for determining to what extent Miocene aridification affected the group’s diversification.

### Biogeographic Patterns in Peripatidae

The initial diversification of Neopatida (our only sampled lineage within Peripatidae) dated to the Upper Triassic ca. 214 Ma (95% HPD: 130–306 Ma). This corresponded to the divergence between *Oroperipatus* and the rest of Neopatida. Divergences between major groups in the Eastern clade were estimated to have occurred in quick succession in the mid-Cretaceous, with mean ages ranging between 97 and 126 Ma (95% HPD inclusive of all backbone nodes: 47–191 Ma). The timing of the Eastern clade’s diversification corresponds closely with the results of Giribet et al. (2018) using Sanger markers (70∼120 Ma). This rapid cladogenesis further bolsters the hypothesis that the Neotropical peripatids represent true evolutionary radiation, in accordance with the extensive gene tree conflict apparent in the SuperQ network (supplementary fig. S32, Supplementary Material online) and the extremely short and poorly supported internodes in the ASTRAL trees of Peripatidae (fig. 3*c*).

With an estimated diversification time of ∼214 Ma, Neopatida represents an ancient lineage in the Neotropics, particularly compared to much of the vertebrate fauna (Hedges 2006). This old age implies that onychophorans survived the K–Pg extinction *in situ*, despite the proximity of the bolide impact near the Yucatán Peninsula and its resulting indirect effects (e.g., debris clouds, tsunamis) (Maurrasse and Sen 1991). It is possible that their restriction to sheltered, often subterranean microhabitats such as leaf litter, rotting logs, and caves facilitated their survival. Indeed, there are several examples of Neotropical soil animals that persisted across the K–Pg boundary, such as hooded tick spiders (Fernández and Giribet 2015), mite harvestmen (Benavides et al. 2019), and caecilians (Pyron 2014). Unfortunately, the current understanding of Neotropical onychophoran biodiversity is highly incomplete (Oliveira, Read, et al. 2012) and the lack of a proper taxonomic and systematic framework for the group precludes testing macroevolutionary hypotheses, such as whether Neopatida’s diversification rate through time contains signatures of the end-Cretaceous extinction, using modern comparative methods (e.g., Rabosky 2014; May et al. 2016). Future work should continue to focus on the description of new species using integrative taxonomy (e.g., detailed morphological examination of multiple individuals per population paired with high-throughput molecular data analyzed in a phylogenetic framework) so that such hypotheses may eventually be tested.

Even with those taxonomic limitations, several biogeographic patterns are evident in Neopatida. For one, it is clear that a strict vicariance scenario is insufficient to explain their distribution across the Neotropics. This is obvious from their presence on oceanic islands such as the Galapagos; and while we were unable to include these lineages in our study, they are also known from several islands in the Lesser Antilles, such as Saint Vincent (home to the type species of Onychophora, *Peripatus juliformis*). Additionally, the divergence time between *Oroperipatus* and the other members of Neopatida ca. 214 Ma far predates the formation of a discontinuous Panama volcanic arc ∼73 Ma, much less the continuous land connection between North and South America that formed ∼2.8 Ma (Buchs et al. 2010; O’Dea et al. 2016), and yet *Oroperipatus* is known from Mexico (*O. eisenii*; while our phylogeny only includes representatives of *Oroperipatus* from Mexico and the Galapagos, the genus is mainly found in Ecuador and Colombia). The timing of this divergence is not compatible with any geologically informed vicariance scenario, and instead suggests a history in which a lineage of *Oroperipatus* dispersed, perhaps via a stepping-stone or sweepstakes scenario, to Mexico. A similar stepping-stone scenario has also been proposed to explain the dispersal of land vertebrates between North and South America in the Upper Cretaceous (Ortiz-Juareguizar and Pascual 2011).

Furthermore, our chronogram indicates that peripatids have been diversifying in Puerto Rico since ∼27 Ma (95% HPD: 9–50 Ma), and our phylogenetic analyses indicate that they share common ancestry with taxa from the Guiana Shield and Brazil. Interestingly, taking into account the 95% HPD interval, this reconstructed history concords with the controversial GAARlandia hypothesis (Iturralde-Vinent and MacPhee 1999), which proposes that a semi-continuous walkway existed between 33–35 Ma connecting South America to the Greater Antilles atop the Aves Ridge. This land bridge was proposed as an explanation for the presence of nonvolant vertebrates in the Greater Antilles that were closely related to species from South America. More recently, GAARlandia has been invoked as a possible mechanism for the colonization of the Greater Antilles by relatively dispersal-limited animals including spiders (Chamberland et al. 2018; Tong et al. 2019), scorpions (Esposito and Prendini 2019), and toads (Alonso et al. 2012). However, there is currently scant geologic evidence for a continuous walkway (Ali 2012; Philippon et al. 2020), and indeed the general pattern of movement from South America to the West Indies follows the direction of water currents in the Caribbean Sea, as well as the path taken by nearly all hurricanes in the region (Hedges 2006). While we cannot rule for or against the possibility of colonization via GAARlandia with our chronogram, we note that we were unable to include samples from other Caribbean islands (e.g., Jamaica, Hispaniola), and as such our results may change with denser taxonomic sampling. Another plausible mechanism for the presence of peripatids on Caribbean islands is rafting via a sweepstakes scenario, possibly as a result of tropical storms, a scenario proposed by Monge-Nájera (1995).

Finally, the uplift of the Andes is well-understood to have affected diversification rates and distribution patterns in many groups endemic to the Neotropics (e.g., Antonelli et al. 2009; Elias et al. 2009; McGuire et al. 2014; Lagomarsino et al. 2016). Given that Neopatida is estimated to have started diversifying in the Late Triassic, it is therefore likely that Andean orogeny, which started in the Jurassic or Cretaceous and continues to the present (Chen et al. 2019), likely shaped the diversification of Neopatida. This is perhaps most evident in the initial divergence between *Oroperipatus*, which is found west of the Andes, and the rest of Neopatida, which predominantly is found east of the Andes. But again, in the absence of a well-characterized taxonomic framework, a dense sampling of Andean taxa, and a comprehensive understanding of their geographic range, we cannot draw any pointed conclusions about whether or not Andean orogeny affected cladogenesis or extinction rates in the group.

### Relative Dispersal Abilities in Peripatidae and Peripatopsidae

There are multiple places in the phylogeny of Peripatopsidae that reflect a history of Gondwanan vicariance, and indeed there is little evidence of *trans*-oceanic dispersal in the family. This fact has contributed to Onychophora being heralded as a short-range endemic taxon, characterized by low dispersal abilities and high population structure (Harvey 2002). But within Neopatida, over-water dispersal––at least over moderately short distances––seems to have occurred multiple times to oceanic Caribbean islands and probably to the Galapagos (although some have argued that the occurrence of the Galapagos species is a human introduction, no one has demonstrated it [Espinasa et al. 2015]). This discrepancy is striking and begs for a biological explanation, which remains elusive given the generally limited understanding of many basic aspects of onychophoran biology, such as survival ability in sea water (see Monge-Nájera et al. 1993; Monge-Nájera 1995).

One possible avenue of future interrogation, though, could be reproductive mode, which is surprisingly variable across the phylum (fig. 2). Neopatida is the only lineage within Onychophora that utilizes placentotrophic viviparity, characterized by small, yolkless eggs that receive nourishment exclusively from the mother via a “placenta.” In contrast, members of Peripatopsidae have a variety of reproductive modes, including oviparity, matrotrophic viviparity, lecithotrophic viviparity, and a combination of matrotrophic and lecithotrophic viviparity (Mayer et al. 2015; Oliveira et al. 2018). Even within Peripatidae, the Southeast Asian genus *Eoperipatus* exhibits lecithotrophic viviparity (though the reproductive mode of the West African genus *Mesoperipatus* is unknown beyond “viviparity”).

All modes of viviparity necessitate increased maternal investment in offspring development compared to oviparity, which Monge-Nájera (1995) hypothesized could be a result of evolutionary pressures from parasites or predators. However, a female onychophoran with multiple internally developing embryos at different developmental stages that survived a period of over-water rafting, perhaps in a rotting log or vegetative mat, may hypothetically be able to establish a population upon reaching an island. Placentotrophic viviparity could therefore be an exaptation that allowed neopatids to colonize oceanic islands after a period of rafting. Furthermore, peripatid females have been shown to mate when very young and subsequently retain sperm long-term in seminal receptacles for later fertilization, another trait that could aid in the establishment of new populations; by contrast, the seminal receptacles of peripatopsid females are believed to function only as short-term sperm stores (Anderson and Manton 1972; Mayer 2007). Future research into the ecological tolerances of different onychophoran species, characterization of the relative contribution of maternal investment in species with different reproductive modes, and a more comprehensive resolved phylogeny of Neopatida that clarifies exactly how many lineages have been established as a result of over-water dispersal will all be useful in testing this hypothesis.

## Conclusions

Onychophora is an ancient phylum that retains biogeographic signatures of many major events in Earth’s history, from the breakup of Pangaea and Gondwana to a putative radiation in the Neotropics during the Cretaceous. We also recovered contrasting biogeographic patterns between Peripatopsidae and Peripatidae that reflect different dispersal capabilities in the two families and propose that divergent life-history strategies and reproductive modes may be related to these differential abilities. Furthermore, we showed that phylogenomic analysis of transcriptomic data is a promising avenue for future taxonomic and systematic work in the group, as it was able to resolve several previously unknown relationships, particularly in Neopatida, when analyzed at the nucleotide (instead of AA) level. Future studies could also use the transcriptomes generated in this study to aid in the design of a target enrichment probe set, which would allow the use of ethanol-preserved museum specimens to expand sampling and improve taxonomy by including types into future data sets. However, rogue behavior of specific taxa paired with the rapid cladogenesis of the Neotropical species may reflect a polytomy, a scenario with thorny taxonomic implications. Currently, Neopatida comprises a handful of “catch-all” genera (e.g., *Peripatus*, *Epiperipatus*, *Macroperipatus*), and series of monotypic genera (e.g., *Plicatoperipatus*, *Principapillatus*, *Speleoperipatus*), all of which have questionable validity (Giribet et al. 2018; Costa et al. 2021, this study). In their paper, Giribet et al. (2018) proposed, but did not enact, a drastic solution—reverting to the classification scheme of (Bouvier 1899a, 1899b) by dividing Neopatida into two genera, one corresponding to the “Péripates andicoles,” that is, *Oroperipatus*, and placing the rest of the taxa into a second, highly diverse genus corresponding to the “Péripates caraïbes.” While we likewise do not advocate for this taxonomic change, the results of our analyses underscore the flaws of the current system (see also Costa et al. 2021), and suggest that the work of erecting new, valid genera will require more extensive and informative sequence data beyond a handful of Sanger sequencing loci, as well as a thorough reexamination of morphology, karyotype, genomes, and/or behavior to find synapomorphies for phylogenetic groups.

## Materials and Methods

### Sample Collection and Molecular Methods

Specimens of 25 onychophorans, representing eight peripatopsids and 17 peripatids, were newly sequenced for this study. Additionally, we included six onychophorans from previously published studies, all of which were downloaded from the NCBI Sequence Read Archive (SRA) (supplementary table S1, Supplementary Material online). Sampling within Peripatopsidae covered all major landmasses from which they are known, with the exception of New Guinea (and some outlying islands) and Tasmania. Sampling within Peripatidae was restricted to the Neotropics, as we did not have RNA-quality material from the monotypic genus *Mesoperipatus* from West Africa or from the Southeast Asian genera *Typhloperipatus* (monotypic) and *Eoperipatus* (five described species). Ecdysozoan outgroup transcriptomes were downloaded as raw data from SRA or as assemblies in Dryad (supplementary table S2, Supplementary Material online).

Specimens were initially preserved in RNA*later* and later flash-frozen in liquid nitrogen and stored at –80ºC. All newly sequenced specimens from this study are deposited in the Invertebrate Zoology collection of the Museum of Comparative Zoology (MCZ), Harvard University and can be accessioned through the online portal MCZbase (https://mczbase.mcz.harvard.edu). Illumina sequencing of transcriptomes followed the protocols of Baker et al. (2020), with all samples sequenced as 150 bp paired-end reads on an Illumina HiSeq 2500 at the Bauer Core Facility at Harvard University. New sequences are deposited in SRA (BioProject PRJNA694953).

### Data Sanitation and Orthogroup Inference

Quality filtering, trimming of reads, and strand-specific assembly of transcriptomes followed the methods of Baker et al. (2020). mRNA contigs were translated from nucleotide sequences to amino acid sequences with TransDecoder v 3.0 (Haas et al. 2013) and predicted peptide (.pep) files were filtered to select only one peptide per putative gene by choosing the longest open reading frame (ORF) using a Python script from Laumer et al. (2015). Due to limited phylogenetic signal within Neopatida, we also ran additional analyses for this clade using the coding sequences (.cds) produced in TransDecoder, again selecting only one sequence per orthogroup by choosing the longest ORF. The completeness of each assembly was evaluated with BUSCO by comparison with the Metazoa database (Simão et al. 2015).

Orthologous genes were predicted across all samples, for both amino acid and codon data sets, using the all-by-all graph-based clustering method Orthologous Matrix Algorithm, OMA standalone v 2.2.0 (Altenhoff et al. 2018). Each OMA-generated amino acid (AA) orthogroup was aligned individually with MAFFT v 7.309 (Katoh and Standley 2014). To reduce alignment uncertainty, we also ran Zorro (Wu et al. 2012) on each aligned orthogroup, discarding all sites with a probability score below 5. For the codon-level orthogroups, each locus was aligned individually using the reading frame-aware aligner MACSE v 2.03 (Ranwez et al. 2011), specifying the standard genetic code and discarding all stop codons at alignment ends. Orthogroups that contained frameshifts (*n* = 10) or that were invariant (*n* = 3) were excluded from downstream analyses. To reduce alignment uncertainty of the codon-level orthogroups, we used the trimAlignment function of MACSE to remove alignment ends until at least 30% of sequences included nucleotide data at that site.

### Matrix Construction

We constructed a series of data matrices to accommodate different potential systematic biases (fig. 2). M1 and M2 corresponded to amino acid orthogroup matrices containing all onychophorans and outgroup taxa with 50% and 75% minimum taxon occupancy, respectively. Taxon occupancy was defined as the orthogroups present in at least that percentage of taxa. To minimize the potential effect of long-branch attraction, we created M3 and M4 by removing all non-onychophoran taxa and including orthogroups with 50% and 75% taxon occupancy, respectively. Because of the limited phylogenetic signal in our data for Peripatidae––something also seen with earlier Sanger-based data––we also constructed matrices using the untranslated amino acid sequences from TransDecoder. M5 and M6 contained only the samples in Peripatidae (i.e., the Neotropical specimens) and orthogroups with 50% and 75% taxon occupancy, respectively. We further refined M5 by performing a chi-square test of compositional homogeneity in the program BaCoCa (Kuck and Struck 2014) and removing all loci with a *p*-value <0.99 (M7). Finally, we removed the fastest- and slowest-evolving 20% of loci from M5 so as to account for the potential biasing effect of extreme evolutionary rates (M8). Evolutionary rate was calculated in the program TrimAl (Capella-Gutiérrez et al. 2009). Selected orthogroups were concatenated into matrices using Phyutility (Smith and Dunn 2008) for subsequent phylogenetic analysis.

### Phylogenetic Analysis

For all matrices, we performed a series of phylogenetic analyses covering maximum-likelihood (ML), Bayesian inference (BI), and species tree methods. ML tree searches were performed in IQ-TREE-MPI v 1.6.10 (Nguyen et al. 2015). For the amino acid matrices (M1–M4), we used the ML implementation of the Bayesian CAT model, a nonpartitioned analysis that included the 10–60 class profile mixture models, as well as model selection with ModelFinder (Kalyaanamoorthy et al. 2017). The untranslated DNA sequence matrices (M5–M8) were also subjected to model testing, including the nonpartitioned GHOST model to account for heterotachy (Crotty et al. 2020), specifying codon models. For all ML analyses, nodal support was assessed using 1500 ultrafast bootstrap replicates (UFBoot) (Hoang et al. 2018) and 1500 replicates of the SH-like approximate likelihood ratio test (SH-aLRT). BI tree searches were run in ExaBayes 1.5 (Aberer et al. 2014) using two runs of two chains and up to 5 million generations until the average standard deviation of split frequencies was below 5%. A consensus of both runs was created, discarding the first 25% of trees as burn-in. We also used ASTRAL 5.7.5 (Zhang et al. 2018) as a concatenation-free species tree inference method. Individual gene trees for ASTRAL input were generated in RAxML for M1–M4, using the model selection feature PROTGAMMAAUTO, and in IQ-TREE for M5–M8, using ModelFinder for codon models. Finally, we performed a BI analysis on M4 and M6 in PhyloBayes-MPI v 1.7a (Lartillot et al. 2013) using the CAT+GTR site-heterogeneous mixture model. PhyloBayes was only run on these matrices due to computational limitations, as they represent some of the smallest matrices constructed from AA and codon sequence data, respectively. For each matrix, two chains were run in parallel until they reached convergence, assessed using the bpcomp and tracecomp commands, and discarding the first 25% of trees as burn-in.

We also conducted an approximately unbiased (AU) test (Shimodaira 2002) in IQ-TREE using M2 to determine whether the monophyly of South African taxa was significantly better than paraphyly (*Metaperipatus* + *Peripatopsis*), the latter having been previously recovered in Sanger-based studies (e.g., Giribet et al. 2018).

Due to the inconsistent placement of the Amazonian *Epiperipatus* sp. (MCZ-136557), we performed additional analyses to explore the strength of support for different topologies within Neopatida. First, we ran IQ-TREE on M5–M8 omitting this sample to determine how or whether the topologies changed with its exclusion, following the same parameters described above. We also visualized incongruence between the ML gene trees computed in IQ-TREE using the program SuperQ v 1.1 (Grünewald et al. 2013), which breaks down gene trees into quartets and generates “supernetworks” in which edge lengths are scaled according to quartet frequencies (that is, longer lines indicate stronger support for a bipartition across a set of gene trees). We generated a supernetwork for M5 and visualized the resulting NEXUS file in SplitsTree v 4.14.2 (Huson and Bryant 2006), filtering out the two specimens of *Oroperipatus* for visual clarity. Additionally, we performed a quartet likelihood mapping analysis (Strimmer and von Haeseler 1997) in IQ-TREE to interrogate whether the orthogroups in M5 were capable of resolving the position of the specimen from Amazonas. Clusters were defined as follows: 1) Trinidad & Tobago (*Macroperipatus torquatus*, *Epiperipatus trinidadensis*, *Epiperipatus broadwayi*); 2) Amazonas (*Epiperipatus* sp. MCZ-136557); 3) Puerto Rico–Guyana–Brazil (both specimens of *Peripatus juanensis*, *Peripatus* sp. MCZ-46445, *Epiperipatus* sp. MCZ-141131, and *Epiperipatus* sp. MCZ-141132); 4) Central America (*Epiperipatus vagans*, *Epiperipatus* sp. MCZ-49455, *Epiperipatus* sp. MCZ-141126, *Epiperipatus solorzanoi*, *Epiperipatus bernali*, and both specimens of *Macroperipatus valerioi*). All other terminals were ignored, and all available quartets were mapped.

Data sanitation, transcriptome assembly, orthogroup inference, and phylogenetic analyses were run on the Cannon cluster, supported by the FAS Division of Science, Harvard University.

### Molecular Dating

To estimate the timing of divergences within Onychophora and interpret its biogeographic history, we performed a molecular dating analysis with the PAML v4.9 (Yang 2007) programs codeml and MCMCTree, implementing the approximate likelihood method (dos Reis and Yang 2011). We used the topology recovered by IQ-TREE analysis of M2 as our fixed input tree. We also used the M2 alignment to infer branch lengths, as it was the smallest and most complete matrix that included ecdysozoan outgroups (where we placed several calibrations based on fossil specimen ages) and would minimize the effect of missing data on branch length inference. Hessian matrices were calculated with codeml using empirical amino acid frequencies and the LG substitution model with five rate categories assuming the gamma distribution across sites, using the default parameters for the Dirichlet–Gamma density and the birth–death process, and the correlated rates model. The resulting matrix was then used as a starting point in divergence time estimation. For each calibration scheme (see below), two independent runs were launched with a burn-in of 500,000, sample frequency of 1,000, and sample size of 20,000 (20 million generations total). Convergence of the runs was assessed in Tracer v 1.7 (Rambaut et al. 2018), and all parameters reached an ESS >200.

We constrained the following outgroup nodes, all of which followed the recommendations of Wolfe et al. (2016): 1) the Priapulida–Nematomorpha split (crown Ecdysozoa) was set to a maximum age of 636 Ma (the maximum age of the Lantian biota) and a minimum age of 529 Ma (the age of strata containing *Rusophycus* trace fossils) under a uniform prior so as to be as uninformative as possible (Tong et al. 2015). 2) The Arthropoda–Onychophora divergence was constrained to a minimum age of 529 Ma. 3) The Mandibulata–Chelicerata split (crown Arthropoda) was set to a minimum age of 514 Ma. 4) The Pycnogonida–Euchelicerata split (Chelicerata) was set to a minimum age of 509 Ma. Additionally, we constrained Peripatidae to a minimum age of 100 Ma, reflecting the age of the peripatid fossil *Cretoperipatus burmiticus* from Burmese amber (Grimaldi et al. 2002; Oliveira et al. 2016). The oldest onychophoran fossil, *Antennipatus montceauensis*, is from the Montceau-Les-Mines Lagerstätte, which dates to the Stephanian (upper-boundary of 299 Ma) (Garwood et al. 2016). However, the fossil lacks any features that would allow one to assign it to stem-group Onychophora, stem-group Peripatidae, stem-group Peripatopsidae, or crown-group Peripatidae or Peripatopsidae. As such, we ran two divergence analyses, one where we conservatively treated the fossil as a stem-group onychophoran and constrained the clade to a minimum age of 300 Ma, and another where we added no calibration on the age of Onychophora.

### Ancestral Character Reconstruction

We performed an ancestral character reconstruction on reproductive mode using the R package “phytools” (Revell 2012) and the chronogram with Onychophora constrained to a minimum of 300 Ma as the input tree. Equal rates, symmetrical, and all-rates-different models were generated, and the AIC was used to select equal rates as the best fitting model.

## Supplementary Material

Supplementary data are available at *Molecular Biology and Evolution* online.

## Author Contributions

C.M.B., R.S.B-Y., C.S.C., and G.G. conceived of the study and collected and identified specimens. C.M.B. carried out laboratory work, analysed the data, and drafted the manuscript. All authors edited the manuscript and gave final approval for publication.

## Data Availability

New sequences are deposited in SRA (BioProject PRJNA694953, SRA SRR13614423–SRR13614447). An electronic supplement (supplementary figs. S1–S33 and tables S1–S3, Supplementary Material online) as well as all Nexus alignments, concatenated and gene tree Newick files, the likelihood mapping output, and the SuperQ network are deposited in Harvard Dataverse: https://doi.org/10.7910/DVN/OGIZFP

## Permits

All specimens acquired by the authors for this project were collected under valid permits (New Zealand multiple permits [38002-RES]; Australia [QLD #WITK00845202; WA Permits #OF000190, #CE000648, #SF004565]; Chile [Autorización #026/2014]; South Africa [Eastern Cape permits #CRO 108/11CR and CRO 109/11CR; KZN #OP 4085/2011; Western Cape Permit #AAA007-00344-0035]; Puerto Rico [#2015-IC-056]; Trinidad & Tobago [Trinidad permit A No. 1176; B No. 000573; Tobago Special Game License without number]; Brazil [#005/2020 to Alessandro Ponce de Leão Giupponi]).

## Supplementary Material

msab251_Supplementary_Data
