# Supplementary material for: Phylogenomic Analysis of Velvet Worms (Onychophora) Uncovers an Evolutionary Radiation in the Neotropics
Source: Mol Biol Evol. 2021 Aug 24;38(12):5391–404. doi: 10.1093/molbev/msab251 (PMC8662635; doi:10.1093/molbev/msab251)
Supplement: msab251_Supplementary_Data [file msab251_supplementary_data.docx]

**Supplementary Information for**

**Phylogenomic analysis of velvet worms (Onychophora) uncovers an evolutionary radiation in the Neotropics**

Caitlin M. Baker^1,2^, Rebecca S. Buckman-Young^1^, Cristiano S. Costa^3^, and Gonzalo Giribet^1^

^1^ Museum of Comparative Zoology, Department of Organismic and Evolutionary Biology, Harvard University, Cambridge, MA, USA 02138

^2^ Current address: Department of Integrative Biology, University of Wisconsin–Madison, Madison, WI, USA 53706

^3^ Laboratório de Sistemática e Taxonomia de Artrópodes Terrestres, Departamento de Biologia e Zoologia, Instituto de Biociências, Universidade Federal de Mato Grosso. Avenida Fernando Correa da Costa, 2367, Boa Esperança, CEP-78060-900, Cuiabá, MT, Brazil

**Email:**  cmbaker6@wisc.edu

**Figure S1**. Phylogeny inferred from IQ-TREE analysis of M1. Asterisks (*) mark nodes with full support; remaining nodes show the Shimodaira-Hasegawa approximate likelihood ratio test and ultrafast bootstrap support values.

**Figure S2**. Phylogeny inferred from ASTRAL analysis of M1. Asterisks (*) mark nodes with full support; remaining nodes show local posterior probabilities.

**Figure S3**. Phylogeny inferred from ExaBayes analysis of M1. Asterisks (*) mark nodes with full support; remaining nodes show the posterior probability.

**Figure S4**. Phylogeny inferred from IQ-TREE analysis of M2. Asterisks (*) mark nodes with full support; remaining nodes show the Shimodaira-Hasegawa approximate likelihood ratio test and ultrafast bootstrap support values.

**Figure S5**. Phylogeny inferred from ASTRAL analysis of M2. Asterisks (*) mark nodes with full support; remaining nodes show local posterior probabilities.

**Figure S6**. Phylogeny inferred from ExaBayes analysis of M2. Asterisks (*) mark nodes with full support.

**Figure S7**. Phylogeny inferred from IQ-TREE analysis of M3. Asterisks (*) mark nodes with full support; remaining nodes show the Shimodaira-Hasegawa approximate likelihood ratio test and ultrafast bootstrap support values.

**Figure S8**. Phylogeny inferred from ASTRAL analysis of M3. Asterisks (*) mark nodes with full support; remaining nodes show local posterior probabilities.

**Figure S9**. Phylogeny inferred from ExaBayes analysis of M3. Asterisks (*) mark nodes with full support.

**Figure S10**. Phylogeny inferred from IQ-TREE analysis of M4. Asterisks (*) mark nodes with full support; remaining nodes show the Shimodaira-Hasegawa approximate likelihood ratio test and ultrafast bootstrap support values.

**Figure S11**. Phylogeny inferred from ASTRAL analysis of M4. Asterisks (*) mark nodes with full support; remaining nodes show local posterior probabilities.

**Figure S12**. Phylogeny inferred from ExaBayes analysis of M4. Asterisks (*) mark nodes with full support; remaining nodes show the posterior probability.

**Figure S13**. Phylogeny inferred from PhyloBayes analysis of M4. Asterisks (*) mark nodes with full support; remaining nodes show the posterior probability.

**Figure S14**. Phylogeny of Neopatida inferred from IQ-TREE analysis of M5. Asterisks (*) mark nodes with full support; remaining nodes show the Shimodaira-Hasegawa approximate likelihood ratio test and ultrafast bootstrap support values.

**Figure S15**. Phylogeny of Neopatida inferred from ASTRAL analysis of M5. Asterisks (*) mark nodes with full support; remaining nodes show local posterior probabilities.

**Figure S16**. Phylogeny of Neopatida inferred from ExaBayes analysis of M5. Asterisks (*) mark nodes with full support.

**Figure S17**. Phylogeny inferred from IQ-TREE analysis of M5, excluding *Epiperipatus* sp. Amazonas (MCZ-136557). Asterisks (*) mark nodes with full support; remaining nodes show the Shimodaira-Hasegawa approximate likelihood ratio test and ultrafast bootstrap support values.

**Figure S18**. Phylogeny of Neopatida inferred from IQ-TREE analysis of M6. Asterisks (*) mark nodes with full support; remaining nodes show the Shimodaira-Hasegawa approximate likelihood ratio test and ultrafast bootstrap support values.

**Figure S19**. Phylogeny of Neopatida inferred from ASTRAL analysis of M6. Asterisks (*) mark nodes with full support; remaining nodes show local posterior probabilities.

**Figure S20**. Phylogeny of Neopatida inferred from ExaBayes analysis of M6. Asterisks (*) mark nodes with full support; remaining nodes show the posterior probability.

**Figure S21**. Phylogeny inferred from PhyloBayes analysis of M6. Asterisks (*) mark nodes with full support; remaining nodes show the posterior probability.

**Figure S22**. Phylogeny inferred from IQ-TREE analysis of M6, excluding *Epiperipatus* sp. Amazonas (MCZ-136557). Asterisks (*) mark nodes with full support; remaining nodes show the Shimodaira-Hasegawa approximate likelihood ratio test and ultrafast bootstrap support values.

**Figure S23**. Phylogeny of Neopatida inferred from IQ-TREE analysis of M7. Asterisks (*) mark nodes with full support; remaining nodes show the Shimodaira-Hasegawa approximate likelihood ratio test and ultrafast bootstrap support values.

**Figure S24**. Phylogeny of Neopatida inferred from ASTRAL analysis of M7. Asterisks (*) mark nodes with full support; remaining nodes show local posterior probabilities.

**Figure S25**. Phylogeny of Neopatida inferred from ExaBayes analysis of M7. Asterisks (*) mark nodes with full support.

**Figure S26**. Phylogeny inferred from IQ-TREE analysis of M7, excluding *Epiperipatus* sp. Amazonas (MCZ-136557). Asterisks (*) mark nodes with full support.

**Figure S27**. Phylogeny of Neopatida inferred from IQ-TREE analysis of M8. Asterisks (*) mark nodes with full support; remaining nodes show the Shimodaira-Hasegawa approximate likelihood ratio test and ultrafast bootstrap support values.

**Figure S28**. Phylogeny of Neopatida inferred from ASTRAL analysis of M8. Asterisks (*) mark nodes with full support; remaining nodes show local posterior probabilities.

**Figure S29**. Phylogeny of Neopatida inferred from ExaBayes analysis of M8. Asterisks (*) mark nodes with full support.

**Figure S30**. Phylogeny inferred from IQ-TREE analysis of M8, excluding *Epiperipatus* sp. Amazonas (MCZ-136557). Asterisks (*) mark nodes with full support; remaining nodes show the Shimodaira-Hasegawa approximate likelihood ratio test and ultrafast bootstrap support values.

**
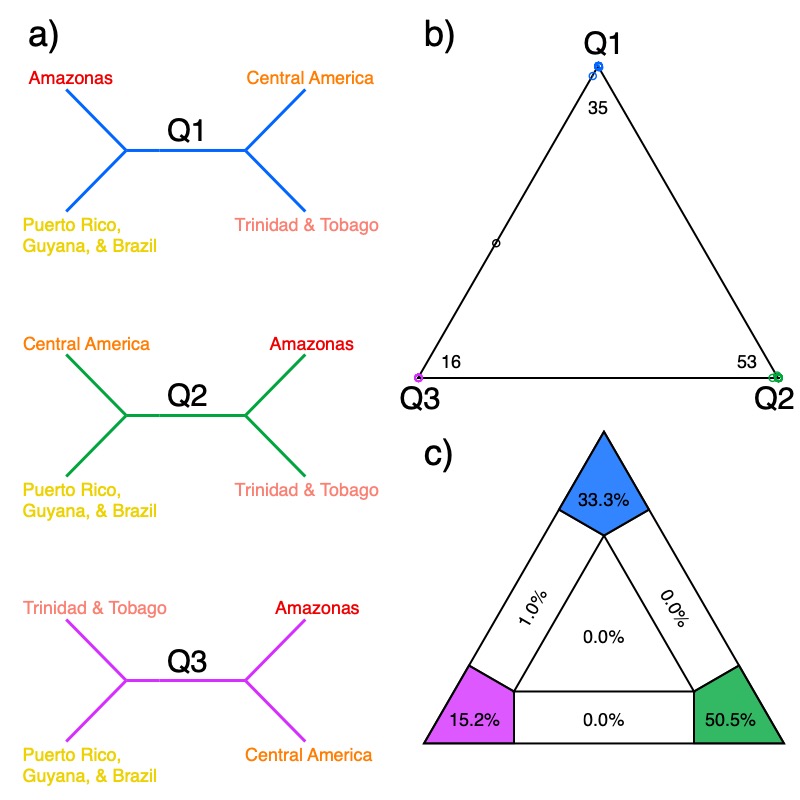
**

**Figure S31**. Results of the quartet likelihood mapping analysis performed on M5. (a) The three possible topologies for our four predefined groups from the Neotropics. (b) Number of quartets that support each of the three possible topologies. Points coloured by which topology they support, coloured as in (a). (c) Percentage of quartets that support each of the possible topologies, with regions of the graph differentiated according to how well a quartet falling in that region can answer a phylogenetic hypothesis.

**Figure S32**. Quartet supernetwork built from 1,147 individual gene trees (M5) in SuperQ, showing conflicting signal between genes. *Oroperipatus eisenii* and *Oroperipatus* sp. Galapagos filtered out for visual clarity. Edges scaled according to bipartition frequency, with specific edges coloured according to clade identity as in main text Figs. 1-3.

**Figure S33**. Chronogram inferred in MCMCTree, specifying no constraint on the age of Onychophora. Numbers at nodes show mean node ages, bars show 95% HPD of age estimates.

**Table S1**. Collection and accession information for onychophoran specimens used in this study. Newly sequenced samples are shown in bold. M3 refers to matrix 3 (see Table S3). BUSCO completeness scores are for the Metazoa database.

| Species | MCZ IZ Catalog # | SRA number | Country | BUSCO | Loci in M3 | Latitude | Longitude | Collection date | Source |
| --- | --- | --- | --- | --- | --- | --- | --- | --- | --- |
| Family Peripatidae | |  |  |  |  |  |  |  |  |
| *Epiperipatus* sp. | 136557 | SRR8627713 | Brazil (Amazonas) | 38.45% | 993 | -2.9295 | -59.9756 | 18-Dec-2013 | (Laumer et al. 2019) |
| ***Epiperipatus* sp.** | **141132** | **SRR13614443** | **Brazil (Mato Grosso)** | **42.02%** | **844** | **-15.4407** | **-55.7704** | **23-Dec-2014** | **this study** |
| ***Epiperipatus* sp.** | **141131** | **SRR13614439** | **Brazil (Rio de Janeiro)** | **42.84%** | **947** | **-22.4588** | **-42.6534** | **21-Dec-2014** | **this study** |
| **Peripatidae sp.** | **141458** | **SRR13614444** | **Colombia** | **68.61%** | **1016** | **5.8806** | **-73.0640** | **4-Dec-2016** | **this study** |
| ***Macroperipatus valerioi*** | **130841** | **SRR13614428** | **Costa Rica** | **39.78%** | **962** | **9.4885** | **-83.9568** | **13-Jul-2015** | **this study** |
| ***Macroperipatus valerioi*** | **130842** | **SRR13614427** | **Costa Rica** | **14.83%** | **436** | **9.4838** | **-83.9396** | **8-Jul-2015** | **this study** |
| ***Epiperipatus solorzanoi*** | **130840** | **SRR13614434** | **Costa Rica** | **40.18%** | **829** | **9.6731** | **-83.0243** | **14-Jun-2015** | **this study** |
| ***Oroperipatus* sp.** | **133614** | **SRR13614445** | **Galapagos** | **9.82%** | **410** | **-0.6249** | **-90.3855** | **Feb-2016** | **this study** |
| ***Peripatus* sp.** | **46445** | **SRR13614437** | **Guyana** | **10.53%** | **617** | **4.1514** | **-58.2146** | **22-Sep-2014** | **this study** |
| ***Oroperipatus eisenii*** | **74293** | **SRR13614446** | **Mexico** | **46.63%** | **836** | **21.4794** | **-105.0776** | **Nov-2015** | **this study** |
| ***Epiperipatus* sp.** | **49455** | **SRR13614425** | **Panama** | **14.01%** | **725** | **8.7916** | **-78.4524** | **19-Jan-2015** | **this study** |
| *Epiperipatus* sp. | 141126 | SRR8320992 | Panama | 77.61% | 936 | 8.7467 | -82.4189 | 9-Sep-2014 | (Mapalo et al. 2020) |
| ***Epiperipatus vagans*** | **141130** | **SRR13614432** | **Panama** | **67.48%** | **1046** | **9.0836** | **-79.6632** | **14-Sep-2014** | **this study** |
| ***Epiperipatus bernali*** | **141128** | **SRR13614442** | **Panama** | **64.72%** | **904** | **8.4332** | **-82.4519** | **10-Sep-2014** | **this study** |
| ***Peripatus juanensis*** | **133571** | **SRR13614436** | **Puerto Rico** | **6.65%** | **579** | **18.1720** | **-67.0445** | **12-Feb-2016** | **this study** |
| ***Peripatus juanensis*** | **133572** | **SRR13614435** | **Puerto Rico** | **9.61%** | **658** | **18.3232** | **-65.8155** | **19-Feb-2016** | **this study** |
| ***Epiperipatus broadwayi*** | **143935** | **SRR13614426** | **Trinidad & Tobago (Tobago)** | **67.69%** | **975** | **11.2869** | **-60.6101** | **26-May-2017** | **this study** |
| ***Epiperipatus trinidadensis*** | **143926** | **SRR13614433** | **Trinidad & Tobago (Trinidad)** | **69.02%** | **1048** | **10.7051** | **-61.2898** | **16-May-2017** | **this study** |
| ***Macroperipatus torquatus*** | **143928** | **SRR13614429** | **Trinidad & Tobago (Trinidad)** | **62.78%** | **1013** | **10.7459** | **-61.2554** | **16-May-2017** | **this study** |
| Family Peripatopsidae | |  |  |  |  |  |  |  |  |
| ***Euperipatoides kanangrensis*** | **131395** | **SRR13614431** | **Australia (NSW)** | **16.87%** | **178** | **-33.9833** | **150.1333** | **––** | **this study** |
| **Peripatopsidae sp.** | **141470** | **SRR13614440** | **Australia (QLD)** | **61.25%** | **898** | **-27.3917** | **152.9236** | **22-Oct-2016** | **this study** |
| **Peripatopsidae sp.** | **141416** | **SRR13614441** | **Australia (QLD)** | **67.28%** | **1008** | **-24.4130** | **151.0387** | **25-May-2015** | **this study** |
| ***Kumbadjena occidentalis*** | **141468** | **SRR13614430** | **Australia (WA)** | **42.43%** | **551** | **-33.9078** | **115.0136** | **10-Jan-2016** | **this study** |
| ***Occiperipatoides gilesii*** | **141469** | **SRR13614423** | **Australia (WA)** | **48.16%** | **846** | **-31.8667** | **116.0667** | **27-Jul-2016** | **this study** |
| ***Metaperipatus inae*** | **138078** | **SRR13614424** | **Chile** | **75.87%** | **1037** | **-38.0163** | **-73.1790** | **13-Nov-2014** | **this study** |
| *Ooperipatellus viridimaculatus* | 29203 | SRR8627697 | New Zealand | 33.23% | 708 | -44.4876 | 168.7874 | 20-Jan-2014 | (Laumer et al. 2019) |
| *Peripatoides aurorbis* | 29204 | SRR8627695 | New Zealand | 47.24% | 867 | -37.8419 | 174.7734 | 10-Jan-2014 | (Laumer et al. 2019) |
| *Opisthopatus kwazululandi* | 131434 | SRR8318947 | South Africa | 82.62% | 1025 | -24.9370 | 31.3752 | 14-Nov-2011 | (Mapalo et al. 2020) |
| ***Opisthopatus highveldi*** | **131328** | **SRR13614447** | **South Africa** | **21.17%** | **257** | **-28.7442** | **31.1376** | **4-Apr-2001** | **this study** |
| ***Peripatopsis bolandi*** | **49527** | **SRR13614438** | **South Africa** | **79.75%** | **1005** | **-34.0047** | **18.9944** | **26-Dec-2014** | **this study** |
| *Peripatopsis overbergiensis* | 131372 | SRR1145776 | South Africa | 51.94% | 963 | -33.9819 | 20.8231 | 17-Apr-2009 | (Sharma et al. 2014) |

**Table S2**. Outgroup taxa used in phylogenetic analysis of M1 and M2.

| **Phylum** | **Species** | **SRA number** | **Source** |
| --- | --- | --- | --- |
| Priapulida | *Priapulus caudatus* | --- | (Kocot et al. 2017) |
| Nematomorpha | *Nectonema munidae* | SRR8618616 | (Laumer et al. 2019) |
| Tardigrada | *Echiniscus testudo* | SAMN10601501–SAMN10601521 | (Mapalo et al. 2020) |
| Tardigrada | *Hypsibius dujardini* | --- | (Yoshida et al. 2017) |
| Arthropoda | *Scutigera coleoptrata* | SRR1158078 | (Fernández et al. 2014) |
| Arthropoda | *Limulus polyphemus* | SRR1145732 | (Sharma et al. 2014) |
| Arthropoda | *Anoplodactylus insignis* | SRR5237777 | (Fernández et al. 2016) |

Table S3. Percentage of loci sampled in Neopatida-specific matrices M5–M8 across taxa. New transcriptomes in bold. See main text for details.

| Species | MCZ Catalog # | Country | M5 % | M6 % | M7 % | M8 % |
| --- | --- | --- | --- | --- | --- | --- |
| ***Oroperipatus eisenii*** | **74293** | **Mexico** | **0.73** | **0.93** | **0.73** | **0.75** |
| ***Oroperipatus* sp.** | **133614** | **Ecuador (Galapagos)** | **0.27** | **0.66** | **0.28** | **0.26** |
| **Peripatidae sp.** | **141458** | **Colombia** | **0.86** | **0.94** | **0.87** | **0.87** |
| ***Epiperipatus trinidadensis*** | **143926** | **Trinidad & Tobago (Trinidad)** | **0.89** | **0.91** | **0.89** | **0.91** |
| ***Epiperipatus broadwayi*** | **143935** | **Trinidad & Tobago (Tobago)** | **0.85** | **0.87** | **0.85** | **0.85** |
| ***Macroperipatus torquatus*** | **143928** | **Trinidad & Tobago (Trinidad)** | **0.87** | **0.91** | **0.87** | **0.88** |
| *Epiperipatus* sp. | 136557 | Brazil (Amazonas) | 0.73 | 0.9 | 0.73 | 0.73 |
| ***Epiperipatus* sp.** | **141131** | **Brazil (Rio de Janeiro)** | **0.75** | **0.93** | **0.76** | **0.76** |
| ***Epiperipatus* sp.** | **141132** | **Brazil (Mato Grosso)** | **0.56** | **0.73** | **0.56** | **0.58** |
| ***Peripatus* sp.** | **46445** | **Guyana** | **0.28** | **0.74** | **0.28** | **0.28** |
| ***Peripatus juanensis*** | **133572** | **Puerto Rico** | **0.32** | **0.79** | **0.32** | **0.3** |
| ***Peripatus juanensis*** | **133571** | **Puerto Rico** | **0.2** | **0.58** | **0.2** | **0.19** |
| *Epiperipatus* sp. | 141126 | Panama | 0.8 | 0.86 | 0.8 | 0.8 |
| ***Epiperipatus vagans*** | **141130** | **Panama** | **0.76** | **0.84** | **0.77** | **0.79** |
| ***Epiperipatus bernali*** | **141128** | **Panama** | **0.78** | **0.82** | **0.79** | **0.78** |
| ***Epiperipatus solorzanoi*** | **130840** | **Costa Rica** | **0.77** | **0.92** | **0.77** | **0.77** |
| ***Macroperipatus valerioi*** | **130841** | **Costa Rica** | **0.78** | **0.91** | **0.78** | **0.79** |
| ***Macroperipatus valerioi*** | **130842** | **Costa Rica** | **0.42** | **0.88** | **0.42** | **0.4** |
| ***Epiperipatus* sp.** | **49455** | **Panama** | **0.39** | **0.8** | **0.39** | **0.37** |

**References**

Fernández R, Edgecombe GD, Giribet G. 2016. Exploring phylogenetic relationships within Myriapoda and the effects of matrix composition and occupancy on phylogenomic reconstruction. *Syst Biol* 65:871–889.

Fernández R, Laumer CE, Vahtera V, Libro S, Kaluziak S, Sharma PP, Perez-Porro AR, Edgecombe GD, Giribet G. 2014. Evaluating topological conflict in centipede phylogeny using transcriptomic data sets. *Mol Biol Evol* 31:1500–1513.

Kocot KM, Struck TH, Merkel J, Waits DS, Todt C, Brannock PM, Weese DA, Cannon JT, Moroz LL, Lieb B, et al. 2017. Phylogenomics of Lophotrochozoa with consideration of systematic error. *Syst Biol* 66:256–282.

Laumer CE, Fernández R, Lemer S, Combosch D, Kocot KM, Riesgo A, Andrade SCS, Sterrer W, Sorensen MV, Giribet G. 2019. Revisiting metazoan phylogeny with genomic sampling of all phyla. *Proc Royal Soc B* 286:20190831.

Mapalo MA, Arakawa K, Baker CM, Persson DK, Mirano-Bascos D, Giribet G. 2020. The unique antimicrobial recognition and signaling pathways in Tardigrades with a comparison across Ecdysozoa. *G3 (Bethesda)* 10:1137–1148.

Sharma PP, Kaluziak ST, Perez-Porro AR, Gonzalez VL, Hormiga G, Wheeler WC, Giribet G. 2014. Phylogenomic interrogation of Arachnida reveals systemic conflicts in phylogenetic signal. *Mol Biol Evol* 31:2963–2984.

Yoshida Y, Koutsovoulos G, Laetsch DR, Stevens L, Kumar S, Horikawa DD, Ishino K, Komine S, Kunieda T, Tomita M, et al. 2017. Comparative genomics of the tardigrades *Hypsibius dujardini* and *Ramazzottius varieornatus*. *PLOS Biol* 15:e2002266.
